# Promise of G-Quadruplex Structure Binding Ligands as Epigenetic Modifiers with Anti-Cancer Effects

**DOI:** 10.3390/molecules24030582

**Published:** 2019-02-06

**Authors:** Antara Sengupta, Akansha Ganguly, Shantanu Chowdhury

**Affiliations:** 1 Integrative and Functional Biology Unit, CSIR-Institute of Genomics and Integrative Biology, Mathura Road, New Delhi-110025, India; sengupta.antara6@gmail.com (A.S.); akanshaganguly1119@gmail.com (A.G.); 2 Academy of Scientific and Innovative Research, Rafi Marg, New Delhi-110001, India; 3 GNR Knowledge Centre for Genome Informatics, CSIR Institute of Genomics and Integrative Biology, Mathura Road, New Delhi-110025, India

**Keywords:** epigenetics, G4-interacting proteins, dietary G4 structure-binding molecules, histones, chromatin, replication

## Abstract

Evidences from more than three decades of work support the function of non-duplex DNA structures called G-quadruplex (G4) in important processes like transcription and replication. In addition, G4 structures have been studied in connection with DNA base modifications and chromatin/nucleosome arrangements. Recent work, interestingly, shows promise of G4 structures, through interaction with G4 structure-interacting proteins, in epigenetics—in both DNA and histone modification. Epigenetic changes are found to be intricately associated with initiation as well as progression of cancer. Multiple oncogenes have been reported to harbor the G4 structure at regulatory regions. In this context, G4 structure-binding ligands attain significance as molecules with potential to modify the epigenetic state of chromatin. Here, using examples from recent studies we discuss the emerging role of G4 structures in epigenetic modifications and, therefore, the promise of G4 structure-binding ligands in epigenetic therapy.

## 1. Introduction

DNA-protein interactions package genomic DNA into globular chromatin. This along with modifications of nucleic acids—for example, methylation of cytosine residues—that otherwise do not affect the sequence of chromosomal DNA constitute the epigenetic state of the genome [1] Modifications of the epigenetic status are closely associated with several diseases including cancer, neurodegenerative and metabolic disorders and autoimmune diseases [2,3,4,5,6]. Therefore molecules that can alter or ‘correct’ aberrant epigenetic modifications are of importance as therapeutics—and are sometimes called ‘epigenetic drugs’ [5].

**Figure 1:**
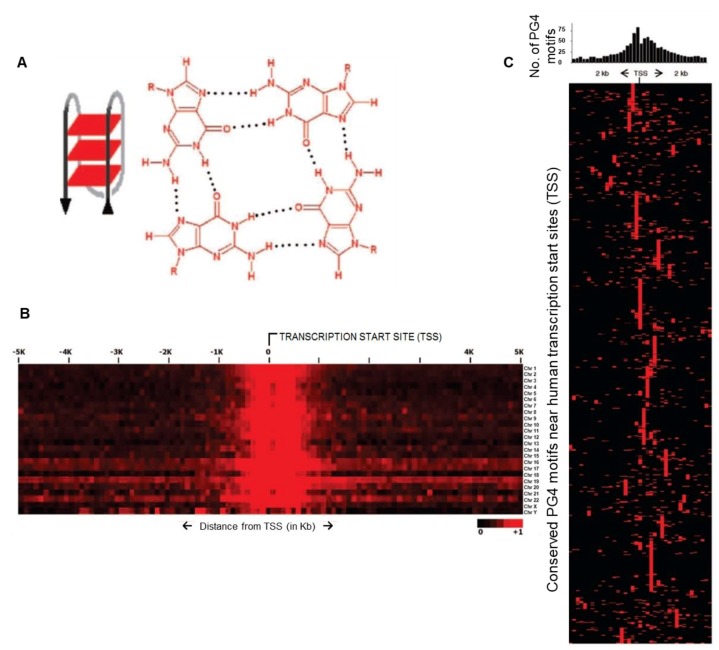
The G4 structure and its relevance. (**A**) G-quadruplex (G4) structural illustration: left panel with G-tetrad planes forming an intramolecular G4 stem, right panel shows Hoogsteen base-pairing of guanines making a G-tetrad. (**B**) Heat map showing averaged relative enrichment of potential G4 (PG4) sequences near TSS across all chromosomes in the human genome (density of PG4s in 100 base windows). (**C**) Heat map of conserved promoter PG4s across organisms: upper panel shows enriched PG4 motifs near TSS, lower panel shows conservation of PG4 motif clusters between human and ‘orthologous’ mouse and rat promoters (red boxes for PG4 motifs per 100 bp window, each row displays individual promoters); 773 human promoters containing 1414 PG4 motifs shown here. Reprinted (adapted) with permission from (Verma, A. et al. Genome-Wide Computational and Expression Analyses Reveal G-Quadruplex DNA Motifs as Conserved cis-Regulatory Elements in Human and Related Species. J. Med. Chem. 51, 5641–5649 (2008)). Copyright (2008) American Chemical Society.

The non-duplex DNA secondary structure called G-quadruplex (commonly called G4), particularly molecules/ligands that specifically interact with G4 structures gain significance in this context. The biological role of G4 structures was first implicated when G-rich telomeric repeats were observed to adopt the four-stranded secondary structure through stacking interactions of guanine-tetrads (Figure 1) [7,8,9,10]. Interestingly, genome-wide analysis revealed a sequence capable of forming G4 structures was enriched in gene regulatory regions (Figure 1) [11,12,13]. This was initially observed through genome-wide analysis in bacteria including *E. coli*—based on which authors proposed a widespread gene regulatory role of G4 structures [11]. Prevalence and conservation within promoters of homologous genes in human, chimpanzee, mouse, and rat further implicated G4 structures in gene regulatory function (Figure 1) [11,14,15]—this was experimentally observed to be so using G4-binding ligands [16]. Gene regulatory functions, and in addition role of G4 structures in replication and recombination have been reviewed earlier [17,18].

The involvement of G4 structures in epigenetic functions, though noted in early work, has received more direct attention in a recent review, where G4 structures have been implicated as structural mediators of epigenetic modifications in chromatin [19]. The authors have focused on human telomerase reverse transcriptase (*hTERT*) promoter transcription factor binding sites and telomerase reactivation in cancer as a case study for epigenetic regulation mediated by G4 structures. Therefore, G4 structure-binding ligands, including ones available as nutrient molecules might be important in epigenetic regulation/modifications—particularly in conditions with established epigenetic aberrations. G4 structure binding ligands have been previously characterized from natural sources (e.g., berberine [20], sanguinarine [21]) and are also artificially synthesized (e.g., ligand 360A [22], pyridostatin [23]). Several of such ligands were found to affect gene regulation through possible epigenetic mechanisms implicated in cancers as well other disorders.

Role of epigenetics in cancer has gained significance as multiple genes and microRNAs related to cancer initiation and progression were reported to exhibit epigenetic abnormalities [24]. Most of these were results of differential regulation of genes coding for epigenetic modifiers itself, leading to silencing of tumor suppressor genes, activation of oncogenes, and altered expression of microRNAs. Several reviews cover this aspect in substantial detail [3,25,26].

Attempts to find out any possible association of G4 structures with genes reported to undergo epigenetic modifications in various cancer types, yielded evidence that several of these gene promoters exhibit potential G4 sequence (PG4), for example, *hTERT* [27], *H19* [28], *KRAS* [29] *BCL-2* [30], *RET* [31,32], *PARP-1* [33,34]. Interestingly, these epigenetic modifications were shown to be regulated by epigenetic modifiers such as DNA methyltransferases (DNMTs) and polycomb group (PcG) proteins, like EZH2, which has been reported to bind to G4 structures in vitro and in vivo respectively [35,36,37] (vide infra). This indicates the possibility that G4 structures could potentially recruit epigenetic modifiers.

With these in mind, we herein focus reviewing literature on how G4 structure-binding molecules and proteins might be important for development of epigenetic therapeutic interventions in future, particularly in cancer.

## 2. G4 Structures Impact Local Chromatin at Telomeric and Extra-Telomeric Sites

### 2.1. The G4 Structure and DNA Base Modifications

**Figure 2:**
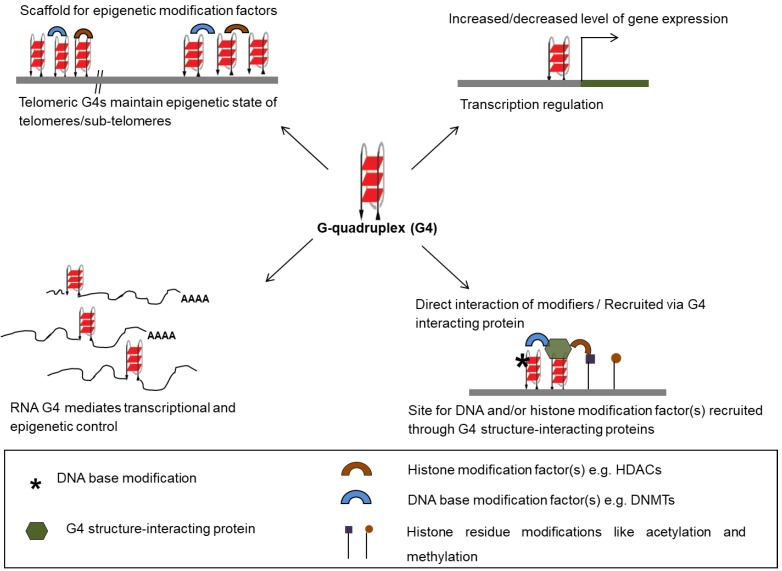
Regulatory roles of G-quadruplex. G4 structures in DNA and RNA are involved in the transcriptional and epigenetic regulation of the genome by acting as anchor sites for recruitment of transcription factors at promoters. G4 structure-interacting proteins recruit epigenetic modifiers upon binding to G4 structures at telomeres and extra-telomeric sites.

Methylation at the C5 position of cytosine within (GGGGCC)8•(GGCCCC)8 repeats—associated with two neurodegenerative diseases amyotrophic lateral sclerosis (ALS) and fronto-temporal dementia (FTD)—was reported to influence stability of G4 structures in the promoter as well as coding region of the *C9orf72* gene [38]. In addition, cytosine methylation of dCGG repeats in the *FMR1* gene, which expand during progression of fragile X-mental retardation syndrome, were reported to result in stabilization of G4 structures formed by the dCGG repeats in vitro [39]. Increased G4 structure stability upon methylation of d(CGG)n oligomers was therefore implicated in repression of *FMR1* in fragile X syndrome [39]. In similar lines, C5 methylation within the G-rich promoter region of the B-cell lymophoma (*BCL-2*) gene, which forms G4 structure, was observed to lead to repression of *BCL-2* known to be abnormally overexpressed in many cancers [30,40]. Results showing that C5 methylation stabilized folding of the G4 structure-forming oligomer further implicated role of DNA methylation-dependent stability of the G4 structure in epigenetic regulation of *BCL-2* [30]. Recently, a CTCF binding site located in the first exon of the human telomerase *hTERT* gene was reported to be disrupted due to the formation of a stable G4 structure following C5 methylation. This was found to result in marked reactivation of *hTERT*—the enzyme essential for telomere synthesis found to over-expressed in more than 90% of human cancers [41,42]. Furthermore, 8-oxoguanine (8oxoG) modification of DNA—from oxidation through reactive oxygen species—was shown to affect stability of promoter G4 structures resulting in altered expression of multiple genes like *c-myc*, *VEGF*, *NTHL1*, and *KRAS* (Figure 2) [29,43,44,45,46].

### 2.2. G4 Structures and Histone Protein Modifications

Recent work further reveal the possible role of the G4 structure in arrangement/modification of histones—proteins required to package chromatin—which help determine the epigenetic state of the genome [47,48,49]. It was noted that the absence of REV1, a helicase that resolves the G4 structure, resulted in replication-associated errors [47]. Because of this, after replication, in cells without REV1 the *β-globin* gene locus was found to lose the K9-dimethylated variant of histone H3 critical for maintaining the repressed state of chromatin. This resulted in de-repression of the *β-globin* gene. The role of the G4 structure was studied further through artificial insertion a G4 structure in the *lysozyme C* gene, which otherwise did not have a G4 structure and therefore was unaffected by the absence of REV1 [47]: artificial insertion of the G4 structure resulted in activation of *lysozyme* expression in cells without REV1. In addition, it was also found that the presence or absence of the G4 structure affected histone H3 modifications (K4-trimethylation and K9/K14-acetylation) at the *BU-1* promoter, which was dependent on the presence of the G4-helicase REV1 [49].

### 2.3. G4 Structures Engage Epigenetic Factors through G4 Binding Proteins

It has been noted that modifications of DNA and histones can cooperate to engage or disrupt binding of regulatory factors [50,51]. Therefore, the role of G4 structures in DNA/histone modifications are expected to impact association of regulatory factors. This was further supported when the binding of epigenetic factors was observed to be dependent on the promoter G4 structure within the cyclin-dependent kinase p21 and telomerase (*hTERT*) promoters [22,27]. Interestingly, at the p21 promoter this was through the recently discovered function of the telomeric protein TRF2 as a transcription factor. Recruitment of the epigenetic repressor complex of proteins including REST/co-REST/LSD1 was through TRF2—where TRF2 binding required presence of the p21 promoter G4 structure [22]. Similarly, in another study authors noted that critical histone modifications for hTERT repression in normal adult cells required binding of the metastasis suppressor factor NME2 [27]. Occupancy of NME2 on the *hTERT* promoter depended on the promoter G4 structure—consistent with NME2-G4 association noted earlier [52]—thereby making the hTERT histone modifications and expression G4-dependent (Figure 2) [27].

Furthermore, high-affinity binding of G4 structures with factors that methylate DNA called DNA methyltransferases (DNMT) was reported recently [53]. Along with earlier work implicating association between G4 structures, global DNA methylation and DNMTs these further supports the possible role of G4 structures in epigenetic modifications [35,54].

### 2.4. G4 Structures Formed by RNA: Role in Epigenetic Modifications

Biological role of G4 structures formed by RNA sequences (RNA-G4) in transcription/translation [55,56,57,58], including epigenetic regulation [59] and the potential of RNA-G4 structures as targets for small molecule-based therapies has been reviewed (Figure 2) [60,61]. Mature human microRNAs were recently discovered to contain RNA-G4 structures that were implicated in miRNA-mRNA-based transcriptional regulation [62]. Multiple studies show the telomeric repeat-containing RNA (TERRA), a long non-coding RNA molecule (lncRNA) that forms G4 structures (RNA-G4), to be important in this context [63,64]. Interestingly, it was noted that RNA-G4 structures formed by TERRA bind to lysine-specific histone demethylase1 (LSD-1)—a histone modifier protein—and this catalyzes the removal of methyl groups from histone 3 at lysine 4 and lysine 9 (H3K4/9) in metazoans [65].

### 2.5. Telomeric G4 Structures and Epigenetic Modifiers

Formation of G4 structures by (TTAGGG)_n_ telomeric repeats in vertebrates has been implicated in the activity of the telomere synthesizing protein telomerase [66]. Relatively recent work reveal telomeric G4 structures might be involved in maintaining the chromatin state of the telomeric/subtelomeric regions (Figure 2) [67,68]. RNA-G4 binding proteins like TLS/FUS and EWS bind TERRA as well as telomeric G4 structures forming a ternary RNA-DNA G4 complex [69,70,71,72]. This complex of proteins was observed to recruit the methyltransferase Suv4-20h2, which tri-methylated K20 residues of histone H4 one of the prime histone modifiers at telomeres [72]. In addition, association of TERRA with the G4 structure-binding RGG3 domain of TLS/FUS mediates K9 tri-methylation of histone H3, which is an essential heterochromatin mark at telomeres [73].

Interestingly, interaction of ATRX, an epigenetic modifier of SWI2/SNF2 family, with telomeric G4 structures was shown to be important in maintaining the ‘dynamic’ state of telomeric chromatin in undifferentiated pluripotent cells [74]. Binding of CBX5 (chromobox homolog 5) along with ATRX at telomeres was involved in inducing the repressed chromatin state. At the same time, ATRX bound to TTAGGG repeats interacted with K4 of H3.3 histones imparting features of open chromatin. In differentiated cells telomeres are predominantly in a closed conformation. Therefore the ATRX-G4 interaction mediated cell cycle-specific ‘open/closed’ telomeric state in undifferentiated pluripotent cells appears to be of significance [74].

## 3. Promise of G4 Structure Binding Molecules in Epigenetics Based Therapeutics

### 3.1. G4 Structure Binding Ligands as Potential Modifiers of the Epigenetic State

Epigenetic drugs include compounds that bind to proteins that affect chromatin organization, such as histone methylation/demethylation inhibitors, bromo-domain inhibitors, HAT inhibitors, HDAC inhibitors, and DNA methyltransferase inhibitors: many of which are at different stages of clinical trials as anti-cancer molecules [75,76,77]. Ligand(s) that bind to G4 structures in DNA/RNA and thereby modulate changes in the chromatin in ways described above, therefore, could be of importance as ‘epigenetic modifiers’. With this in mind, in the following sections we focus on G4 structure-binding ligands that could be relevant in epigenetics. The role of G4 ligands as potential anticancer agents and in antiviral therapy through functions other than epigenetic mechanisms have been reviewed earlier [78,79].

Berberine, a plant alkaloid known to bind G4 structures [20,21], was found to induce hypomethylation of the *TP53* promoter leading to apoptosis in the human multiple myeloma U266 cells [80]. In addition, berberine has been shown to down-regulate histone deacetlyases (HDACs) [81]; up-regulate histone acetyltransferases, demethylases, and methyltransferases, resulting in wide spread changes in methylation of lysine K4/K27/K36 of histone H3 (i.e., H3K4me3, H3K27me3, and H3K36me3) [82]; and, interestingly, affect interaction of DNMTs with microRNAs during malignant transformation of colorectal cancer cells [83]. Although G4-berberine interaction was not directly studied, together these studies implicate berberine in epigenetic functions that could be through G4 structures (Table 1). Similarly, sanguinarine, another molecule obtained from plants, that binds the telomeric and *c-myc* promoter G4 structures [21,84] was noted to epigenetically modify chromatin by inducing altered histone methylation [85].

Based on the effect of the G4 structure observed in replication (described above) small molecules derived from modification of the well-known G4 binding ligand pyridine 2,6-dicarboximide (PDC) was screened using the *BU-1* locus in DT-40 chicken cells [105]. This resulted in several ligands (e.g., PDC12, 14, 22, 23, 25, and 40) that induced transcriptional reprogramming of the *BU-1* locus. This was found to be through the loss of trimethylated-K4 of histone H3 (H3K4me3) and interestingly, cytosine methylation in the *BU-1* gene. Together these suggested the role of the G4 structure in re(placement) of histone marks, a hallmark of epigenetic regulation [105].

As mentioned earlier, epigenetic reorganization of the *hTERT* promoter through interaction of NME2 with the *hTERT* promoter G4 structures results in repression of abnormally overexpressed *hTERT* in cancer cells [27]. Prompted by this authors checked several known G4 binding ligands. Many of these like 9A, 9B, and Bis-ANON (acridine based), JD59 (bis-indole carboxamide) and RR110 (pyridostatin based) showed more than 50% reduction in *hTERT* expression, which was shown to be dependent on presence of the *hTERT* promoter G4 structure [27]. In addition to this, several other G4 ligands have been reported to repress *hTERT* expression [106]. These findings could be useful in development of G4 based epigenetic therapeutic interventions for restricting *hTERT* overexpression as seen in cancer cells.

**Figure 3:**
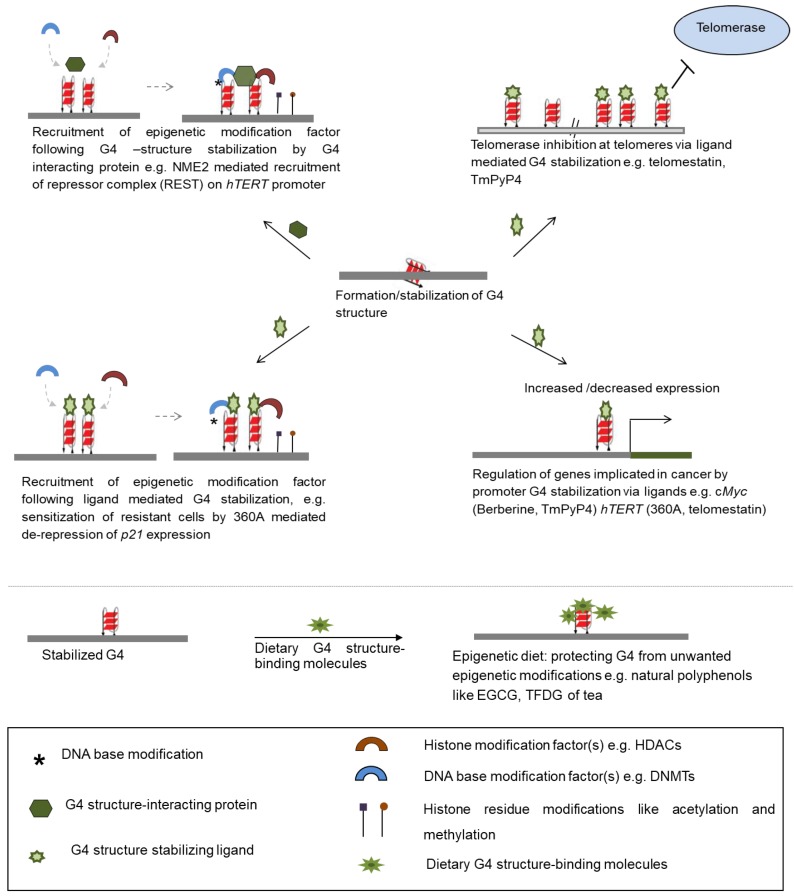
Ways of therapeutic intervention through G4 structure-binding small molecules. Stabilization of G4 structure by means of ligands inhibits telomerase activity at telomeres, regulates expression of genes at transcriptional and epigenetic levels. Proteins stabilizing G4 structure upon binding, allow epigenetic modifiers to dock at the site, further regulating gene expression. Several dietary components protect G4 structure from unwanted modifications by binding to the secondary structure. HDACs: histone deacetlyases; DNMTs: DNA methyltransferases.

Transcription regulation of *p21*—activation of which results in growth arrest of cancer cells on treatment with anticancer drugs—was dependent on TRF2-G4 interactions that induced epigenetic modifications [22]. Anti-cancer drug resistance often results from ineffective *p21* activation [107]. The role of the G4 structure in *p21* epigenetic regulation was tested using the pyridine derivative G4 ligand 360A [22,108]. Authors showed that aggressive MDAMB-231 breast cancer cells, otherwise resistant to the anti-cancer drug doxorubicin, regained doxorubicin-sensitivity in presence of 360A. This was through 360A-mediated de-repression of *p21* in MDAMB-231 cells suggesting the potential function of G4 ligands in modification of cellular epigenetic mechanisms (Figure 3) [22].

### 3.2. Dietary G4 Ligands Can Affect Epigenetic Modifications

Dietary molecules that affect epigenetics and resulting changes in gene regulation include tea polyphenols like ellagic acid [109], epigallocatechin gallate [110], curcumin [111], genistein [112], resveratrol [113], and sulforaphane [114]. Amongst these, epigallocatechin gallate and theaflavin-3,3′-digallate (TFDG) from green tea and black tea, and resveratrol from berries were reported to bind telomeric G4 structures with high affinity [115,116]. Curcumin and ellagic acid were also shown to bind KRAS G4 sequences in vitro [117]. Deficiency of the dietary component folate, a methyl group donor metabolite, was observed to result in global hypomethylation of CpG islands and increased G4 structure formation in HeLa cells [118], consistent with decreased methylation within CpG islands that harbor potential G4 structures in a genome wide study [54]. Berberine was found to impair parasitic infections from *Eimeria* sp. through epigenetic modifications in cells of the gastrointestinal tract in mouse models showing potential as a food supplement in animal husbandry [119]. Furthermore, ROS-induced oxidative stress is known to result in 8-oxo-guanine modifications of Guanine base. As described above such modifications have been reported to affect stability of the G4 structure leading to altered function [44,45,120,121]. Therefore, the effect of dietary anti-oxidants on G4 structures and related epigenetics could be interesting to consider in future (Figure 3).

### 3.3. G4 Structure-Binding Epigenetic Modifier Proteins: Potential for Development of Epigenetic Intervention Agents

Nucleolin, possibly the first protein noted to interact with G-rich oligonucleotides that adopt G4 structure was found to be involved in epigenetic modification of histone H1 implicated in decondensation of chromatin [122,123,124].

Interestingly, in 2009, a metastasis suppressor factor NME2 was found to not only associate with the promoter G4 structure of the oncogene *c-myc* but also important for transcription regulation of *c-myc* suggesting transcription regulatory roles of G4 structures in association with regulatory factors [52]. More recently, NME2 was shown to be involved in epigenetic regulation of *hTERT* through association with the G4 structure in the promoter of *hTERT* [27].

Epigenetic modifiers like DNMT3A and 3B, EZH2, and ATRX (as discussed earlier) bind to G4 structures where epigenetic regulatory functions mediated through such interactions might be of clinical significance [35,37,53,74]. In addition, interaction of TRF2 with G4 structures and/or G-rich binding sites might be important because of epigenetic regulation of genes like *p21* and several other [22], which interestingly was also noted to be dependent on telomere length [125]. The TRF2-mediated epigenetic regulation of *p21* appears to be of added significance in aggressive as well as commonly encountered drug resistant cancer cells.

Somewhat in line with these studies a large scale screening for G4 structure interacting factors using protein microarrays comprising >9000 human proteins found several factors that are involved in binding nucleosomes [126]. It is also likely that function of the G4 structure helicases like FANCJ [127], BLM [128], WRN [129], and REV-1 [49] would be important in epigenetic modifications in a replication-dependent manner (as demonstrated for REV-1) [49]. Similarly factors that bind to RNA G4 structures like the polycomb repressive complex 2 (PRC2) [37], TLS/FUS [72,73], EWS [71], and hnRNP A1 [130,131] suggest further importance of G4 structure-protein interactions in epigenetic regulation [37,71,72,73]. It is of interest to note here that many of the G4 structure interacting proteins possess the positively charged Arg-Gly-Gly (RGG/RG) motif containing domain, which is noted to be important for G4 structure binding (Figure 3) [132,133,134].

## 4. Conclusions and Future Perspectives

For more than a decade G4 structures have been implicated in epigenetic modifications that might impact state of chromatin resulting in altered gene regulation. Recent studies through more direct studies show how G4 structures modify chromatin by not only change in histone and/or DNA modification but also during replication. A growing number of reports suggest that G4 structure has significant role to play in epigenetic control of genes involved in various neurological disorders as well as cancer. Herein we have focused on these studies. This is discussed along with studies that have focused on design and characterization of different classes of small molecule ligands that specifically bind to G4 structures. However, the epigenetic effects of these ligands remain to be confirmed in more physiologically relevant settings, such as in animal models.

Together, these bring forth the promise of the G4 structure binding ligands, including dietary molecules, in affecting epigenetic mechanisms. This becomes particularly notable in cases where changes in epigenetic pattern have been shown to play a role in diseases such as cancer and neurodegenerative disorders. It is possible, therefore, that ligands that bind to G4 structures reinstate/rescue aberrant epigenetic modifications in chromatin and thereby enable therapeutic interventions. Mitochondrial DNA (mtDNA) G4 structures are another promising avenue for small molecule therapeutics; although there is a lack of sufficient data in the field currently mtDNA G4 structures are increasingly under consideration as targets for therapeutic intervention in mitochondrial diseases [135]. A recent study reported that RHPS4, a G4-binding ligand thought to localize to nuclear G4s showed preferential binding to mtDNA G4 structures in both cancerous and non-cancerous cell lines thereby opening new avenues to study mtDNA transcriptional and epigenetic regulation using G4-binding molecules specific to mtDNA [136]. Although intracellular G4 structures are primarily right-handed in orientation, left-handed G-quadruplexes have been observed in vitro [137]; however, there is lack of sufficient evidence to validate their formation by nuclear or mitochondrial G-rich sequences in cellulo. Both left and right handed G4 structures have been shown to form from the same nucleic acid sequence mediated by small molecule binding [138]. Questions about the effect of G4 orientation and ‘handedness’ on genome structural dynamics need to be addressed to improve on the structural sensitivity of G4-binding small molecules.

Although several G4 helicases and G4 binding proteins are known to be associated with genetic diseases not much has been explored for therapeutic interventions. The current arsenal of G4 related therapeutics comprise of G4 selective ligands, which are being attempted to be upgraded to locus specific targeting and G4 DNA aptamers which can bind and inhibit G4 interacting proteins [139]. Aptamers based on promoter G4s are being focused on to serve as G4 decoys in several cases and also being considered as a drug delivery tool as in case of AS1411-drug conjugate nanoparticles [140].

The multitude of data on the biological significance of G4 and G4 structure interacting proteins could also be utilized to design novel drug molecules. Small peptides or peptidomimetics with better stability could be designed to bind and stabilize G4 structures as well as mediate epigenetic changes at locus of interest. This strategy combined with conventional G4 ligands or alone, could be effective in inducing desired epigenetic modification to counter a particular disease state. It could also be specifically delivered to cancer cells using above mentioned aptamer based delivery systems [140]. However, detailed knowledge of protein structure and the interacting G4 structures is still required to develop molecules which can both bind and recruit epigenetic factors. Perhaps tailoring this for a specific locus would be equally important. In conclusion, the G4 structure has been deemed as a promising target in anti-cancer therapy for long now—its emerging role in epigenetic control of pharmacogenes could be a new-found angle in this battle.

## Figures and Tables

**Table 1:** G4 structure binding ligands and their biological roles including in epigenetics.

Ligand	Target G4 Structure(s)	Affected Function/Pathway/Disease	Reference
**Berberine, quindoline**	Telomeric *c-myc* promoter *p53* promoter L-type pyruvate kinase (*L-PK*) promoter	Colorectal cancer, cervical cancer, liver cancer, multiple myeloma, lung cancer Whole genome methylation Non-alcoholic fatty liver disease mediation by increasing *L-PK* expression	[20,80,81,82,83,86,87]
**Telomestatin**	Telomeric *PDGFR-β* promoter, telomerase (*hTERT*) promoter	Inhibition of telomerase activity *PDGFR-β*/*hTERT* downregulation Inhibition of fibroblast development and cellular migration due to hypomethylation of *PDGFR-β* promoter	[88,89,90,91]
**L1H1-7OTD**	*Dele*, *CD6*	Transcriptional regulation	[92]
**Substituted acridines**	*hTERT* promoter, *c-kit* promoter, *KRAS* promoter, telomeric	*hTERT*/*c-kit*/*KRAS* down-regulation Telomere shortening	[27,93,94,95]
**Se2SAP**	*VEGF* promoter	*VEGF* downregulation	[96,97]
**TMPyP4**	miR-1587, *C9orf72* promoter, *UCP1* promoter, *c-myc* promoter, telomeric	Inhibition of miR-1587 regulation of *TAGLN* tumor suppressor gene Amyotrophic lateral sclerosis-fronto-temporal dementia (ALS-FTD) remediation Regulation of fat tissue differentiation *c-myc* transcriptional repression Telomere shortening	[98,99,100,101,102]
**Isaindigotone derivatives**	*c-myc* promoter	Interference of NM23-H2—c-myc promoter binding, c-myc repression	[103]
**Pyridostatin**	Telomeric, *IGFN1* intron	Telomere shortening Change in *IGFN1* mRNA alternative splicing	[23,104]
**Bleomycin**	Telomeric	Telomere shortening	[23]
**PDC12**	*BU-1* promoter	*BU-1* downregulation in chicken DT40 cells	[105]
